# Relationship between polymorphisms in homologous recombination repair genes RAD51 G172T、XRCC2 & XRCC3 and risk of breast cancer: A meta-analysis

**DOI:** 10.3389/fonc.2023.1047336

**Published:** 2023-01-24

**Authors:** Jiayang Yu, Chun-Guang Wang

**Affiliations:** Department of Oncology, Yongchuan Hospital of Chongqing Medical University, Chongqing, China

**Keywords:** breast neoplasms, Rad51 recombinase, single nucleotide polymorphism, DNA repair mechanism mutations, meta-analysis

## Abstract

**Background:**

Genetic variability in DNA double-strand break repair genes such as RAD51 gene and its paralogs XRCC2、XRCC3 may contribute to the occurrence and progression of breast cancer. To obtain a complete evaluation of the above association, we performed a meta-analysis of published studies.

**Methods:**

Electronic databases, including PubMed, EMBASE, Web of Science, and Cochrane Library, were comprehensively searched from inception to September 2022. The Newcastle-Ottawa Scale (NOS) checklist was used to assess all included non-randomized studies. Odds ratios (OR) with 95% confidence intervals (CI) were calculated by STATA 16.0 to assess the strength of the association between single nucleotide polymorphisms (SNPs) in these genes and breast cancer risk. Subsequently, the heterogeneity between studies, sensitivity, and publication bias were performed. We downloaded data from The Cancer Genome Atlas (TCGA) and used univariate and multivariate Cox proportional hazard regression (CPH) models to validate the prognostic value of these related genes in the R software.

**Results:**

The combined results showed that there was a significant correlation between the G172T polymorphism and the susceptibility to breast cancer in the homozygote model (OR= 1.841, 95% CI=1.06–3.21, *P*=0.03). Furthermore, ethnic analysis showed that SNP was associated with the risk of breast cancer in Arab populations in homozygous models (OR=3.52, 95% CI=1.13-11.0, *P*= 0.003). For the XRCC2 R188H polymorphism, no significant association was observed. Regarding polymorphism in XRCC3 T241M, a significantly increased cancer risk was only observed in the allelic genetic model (OR=1.05, 95% CI= 1.00–1.11, *P*=0.04).

**Conclusions:**

In conclusion, this meta-analysis suggests that Rad51 G172T polymorphism is likely associated with an increased risk of breast cancer, significantly in the Arab population. The relationship between the XRCC2 R188H polymorphism and breast cancer was not obvious. And T241M in XRCC3 may be associated with breast cancer risk, especially in the Asian population.

## Introduction

In all countries around the world, cancer is the leading cause of death and an important obstacle to improving life expectancy. Female breast cancer (BC) has overtaken lung cancer as the leading cause of global cancer incidence in 2020, with an estimated 2.3 million new cases, representing 11.7% of all cancer cases (1). The mechanism of breast carcinogenesis is not yet fully understood. It is considered a polygenic disease and has a component of inheritance due to low-penetrant and common genetic variants. The steady repair of DNA damage is very important for the survival of cells and the maintenance of genetic stability (2).

Over the years, it has been increasingly recognized that variations in the genetic background of individuals combined with environmental exposure can ultimately lead to the occurrence and progression of cancer. DNA repair genes have been considered considerable factors in the prevention of genomic damage and continuously monitor chromosomes to correct injuries caused by exogenous agents such as ultraviolet light or endogenous mutagens (3, 4). Aberrant double-stranded break (DSB) repair leads to genomic instability, a hallmark of malignant cells. Double-stranded breaks are repaired by two pathways: homologous recombination (HR) and non-homologous end joining (NHEJ). Previous analysis has revealed several important features of DSB repair in breast cancer cells: (i) HR is evidently increased in breast cancer cells compared with normal cells; (ii) Non-homologous end joining(NHEJ)repair is the major DSB repair route in both normal and malignant breast epithelial cells; (iii) NHEJ efficiency does not differ significantly between normal and cancerous cells (5). The two pathways of DSB repair are independently controlled, and only HR is increased in breast cancer cells compared with normal breast epithelial cells. RAD51 is a homolog of the E. coli RecA protein, which is essential for maintainability such as meiotic and mitotic recombination, and also plays a critical role in homologous recombination repair (HR) of DNA double-strand breaks (DSB) (6–8).

Researchers recently discovered that the Rad51 promoter in cancer cells is on average 840-fold more active in cancer cells than in normal cells and the fusion of RAD51 promoter and diphtheria toxin gene selectively kills cancer cells. Transcriptional targeting therapy using up-regulated HR gene expression can effectively eliminate cancer cells without toxicity to normal tissues. The human RAD51 gene, located on chromosome 15q15.1, is considered to participate in a common DSB repair pathway and is involved in the development of breast cancer development (9). RAD51 functions by assembling on a single-stranded DNA, inducing homologous pairing, and in turn mediates strand invasion and exchange between homologous DNA and damaged site (10). In recent years, the RAD51 gene polymorphism has attracted a great deal of attention. The RAD51 family of genes, including RAD51 and the five RAD51-like genes, are known to have crucial non-redundant roles in this pathway. Recently, researchers have revealed that RAD51 paralogs (RAD51B, RAD51C, RAD51D, XRCC2, XRCC3) could serve as central proteins during the HRR process. The function of RAD51-like genes is to transduce DNA damage signals to effector kinases that promote break repair. A central player in homologous recombination is the RAD51 recombinase that binds to single-stranded DNA at break sites, the XRCC2 and XRCC3 genes are structurally and functionally related to the RAD51 genes (11). Two commonly studied polymorphisms of the RAD51 gene are G135C (rs1801320), a G to C transversion at position +135, and G172T (rs1801321), a G to T transversion at position +172, both of which are located in the 5 Untranslated region (5’UTR) and appear to be related to functional polymorphisms. Two variants of 135G/C and 172G/T would affect mRNA stability or translational efficiency, resulting in altered levels of polypeptide products, altering the function of encoding the RAD51 protein, and in some way influencing DNA repair capacity and malignancies (12). RAD51 interacts with BRCA1 and BRCA2, acting through HR and NHEJ. For example, down-regulation or mutation of DNA DSB repair proteins involved in the NHEJ pathway was shown to be associated with both BC risk and increased chromosomal radiosensitivity (CRS) (13–15). In addition, RAD51 overexpression is acknowledged to be associated with therapeutic antagonism, aggressiveness, metastatic behavior, and poor prognosis.

X-ray repair cross complementing group 2(XRCC2)gene, located in 7q36.1, is an essential part of the homologous recombination repair pathway and a functional candidate for involvement in cancer progression. Its XRCC2 protein product, together with other proteins encoded by the XRCC2 gene such as RAD51L3, forms a complex that plays a critical role in chromosome segregation and the apoptotic response to DSBs (16, 17). As a member of the RAD51 family of proteins, it is widely acknowledged to mediate HRR (18). However, the exact function of SNPs in the XRCC2 gene in response to different DNA-damaging agents still remains unclear. There is a G-to-A polymorphism located in exon 3 of the XRCC2 gene resulting in a substitution of histidine (His) for arginine (Arg). Known as Arg188His (R188H, rs3218536), this polymorphism has been widely investigated to explore its potential impact on cancer susceptibility. Furthermore, DNA damage caused by anticancer drugs and radiation have been documented to require XRCC2 for repair in mammalian cells (19–22). Several pieces of evidence stress that high levels of expression of The X-ray repair cross complementing group 3 (XRCC3), another member of the RAD51 family of proteins, are correlated with radioresistance and cytotoxic resistance in human tumor cell lines, suggesting that XRCC2 could also play a relevant role in the effects of oncotherapy (23–25). XRCC3, as we know, is localized on human chromosomes 14q32.325. A coding SNP (T241M, rs861539) has been reported at the 18,067th nucleotide in exon 7 of the XRCC3 gene, resulting in a substitution of methionine (Met) for threonine(Thr) (25). The XRCC3 protein is involved in the joining of single-strand DNA breaks and the joining of double-strand DNA breaks (26). As a member of the Rad51 DNA repair gene family. It functions in the HRR pathway by repairing double-strand breaks. XRCC3 helps the assembly of the nucleofilament protein and its selection and interaction with the appropriate recombination substrates (12). Likewise, XRCC3 controls HR fidelity and is essential to stabilize heteroduplex DNA in HRR. Furthermore, a mutation in XRCC3 generates severe chromosomal instability. The XRCC2 and XRCC3 genes are necessary for HRR and are required for the formation of RAD51 focus (27, 28). In recent studies, common variants of XRCC2, particularly the encoding SNP of exon 3 (Arg188His), have been identified as potential cancer susceptibility sites, although in this case, the association with breast cancer susceptibility remains unclear. Earlier studies have shown that the XRCC3 Thr241Met polymorphism has long been regarded as a risk factor for many cancers.

We examined whether polymorphisms in these three genes involving homologous recombination with DSB were associated with the risk of breast cancer.

## Materials and methods

### Search strategy and data extraction

All studies investigating the association between polymorphisms in the RAD51 gene and paralog genes, such as the XRCC2 & XRCC3, and the risk of breast cancer, were identified by comprehensive computer-based searches of the PubMed, Embase, Web of Knowledge, and Cochrane Library databases(the last search update on September 2022). The search was carried out using various combinations of keywords such as (‘RAD51 gene’ OR ‘RAD51 recombinase gene’ OR ‘XRCC3 polymorphism’ OR ‘XRCC3 Thr241Met polymorphism’ OR ‘XRCC2’ OR ‘XRCC2 Arg188His polymorphism’) AND (‘polymorphism’ OR ‘variant’ OR ‘variants’).

### Eligibility criteria and selection process

#### Inclusion criteria

Studies included in our meta-analysis needed to have met the following criteria: 1)published in public, full text only; 2) case-control study; 3) sufficient data (genotype distributions for cases and controls) to calculate an odds ratio (OR) with its 95%CI; 4) studies published in English; 5) genotype distribution of the control population consistent with the Hardy-Weinberg Equilibrium (HWE).

#### Data extraction

Two authors independently extracted information from all eligible publications according to the inclusion criteria listed above. Disagreement was resolved by evaluating a third reviewer and discussing until a consensus was reached. The following characteristics were collected from each study: first author, year of publication, country, ethnicity, methods in experiments, source of control groups and genotype frequencies in case and control groups, and the value of HWE. Duplicated primary studies were deleted and only one version of duplicated documents was kept.

#### Data collection

The transcriptome data and clinical information of BC patients were obtained from The Cancer Genome Atlas (TCGA) database (https://cancergenome.nih.gov/). In total, 903 patients with BC were selected from the TCGA cohort. For the transcriptome data from TCGA-BRCA, we download their series files. Some important clinical characteristics including age, pathologic stage (I, II, III, IV, V, and NA), and pathology stage (T, N, M) are available. The datasets listed in **Table 6** are used to discover and verify prognostic factors of BC patients. We assessed the association of each gene with overall survival by univariate and multivariable Cox proportional hazard regression analysis. All statistical tests were two-sided. The Cox proportional hazard model, including several important factors, was employed to estimate the hazard ratio (HR) and 95% CI for each gene for breast cancer survival. We use normalized P values of <0.05 to define statistical significance. This part of statistical tests was performed using the R software.

### Statistical analysis

We first analyze HWE in the controls for each study using a goodness-of-fit test (chi-square or Fisher’s exact test) and the departure of HWE genotype frequency among control subjects was determined by *P <*0.05. Crude odds ratios (OR) with 95% confidence intervals (CI) were used to assess the strength of the association between the RAD51 gene and its paralog polymorphisms and breast cancer susceptibility. The pooled ORs for the RAD51 G172T polymorphism were performed under the dominant model (GG vs. TT+GT), recessive model (TT vs. GG+GT), homozygote model (TT vs. GG), and allelic genetic model (T vs. G). T and G represent the minor and the major alleles, respectively. The same methods were applied to the analysis of other polymorphisms. Stratified analyzes were performed on ethnicity and source of control. A Q-test was performed to assess statistical heterogeneity among studies. The pooled OR was calculated using a fixed effect model if the result of the p-value of the Q test<0.1 indicated significant heterogeneity according to the previous study(Davey and Egger,1997) (29, 30). If the result of the Q test was *P*>0.1, which indicated that the heterogeneity between studies was not significant. Otherwise, a random-effects model was used. Given the potential heterogeneity among studies with different ethnicities and sources of control, the random-effects model was adopted (30). Sensitivity analysis was carried out by removing each study at a time to evaluate the stability of the results under either genotypic models or the allelic model. In addition, the Begg test and Egger’s linear regression test by visual inspection of the funnel plot were carried out to address the potential publication bias, and *P <0.05* was considered an indicator of significant publication bias (30, 31). Cox regression was used to analyze the impact of genes on the prognosis of BC patients and its value in prognostic diagnosis

The Newcastle-Ottawa Scale (NOS) was applied to assess the quality of all studies. The NOS checklist includes three parameters of quality: (i) selected population, (ii) comparability of groups, and (iii) assessment of either the exposure or outcome of interest for case-control studies. The studies scored greater than or equal to 7 were considered to be high quality articles.

## Results

### Studies included in the meta-analysis

**Figure 1:**
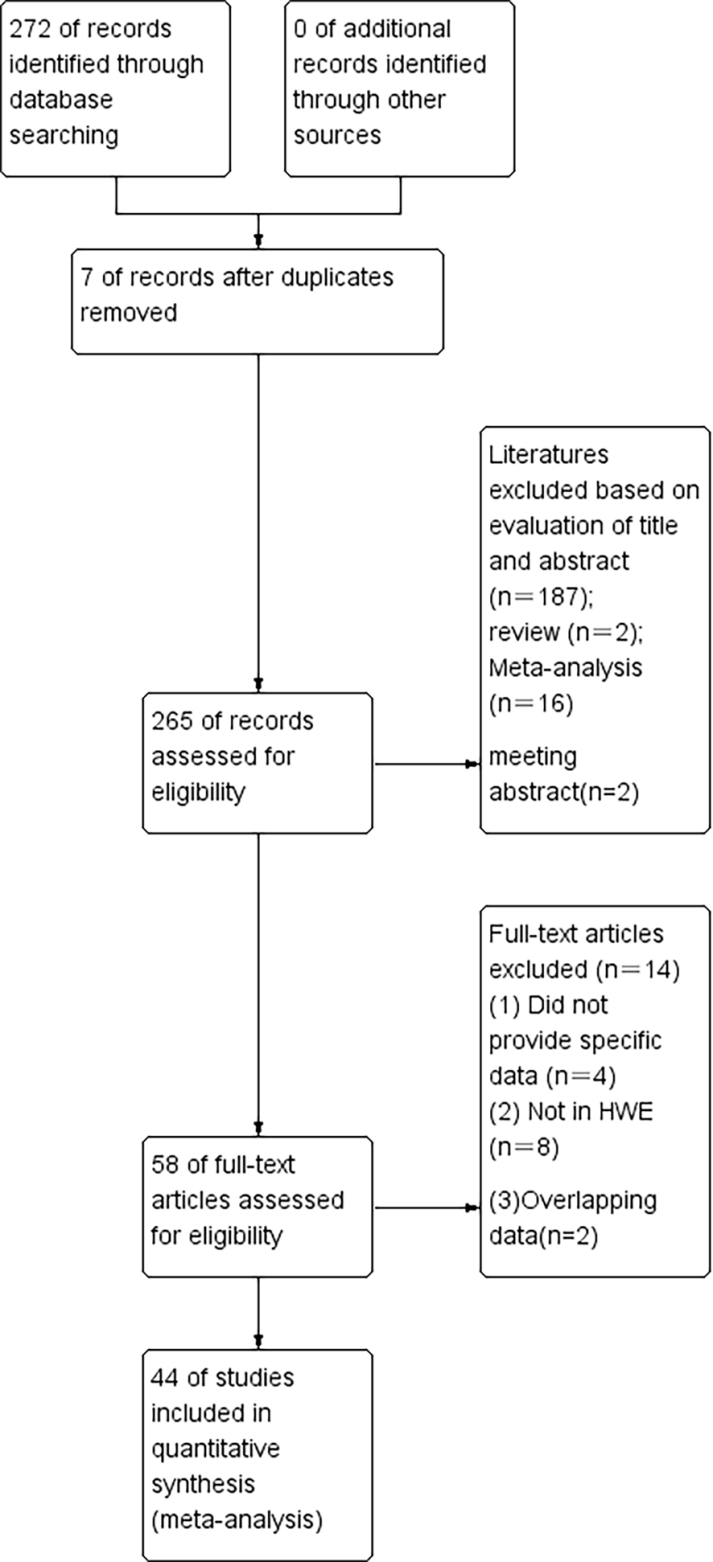
The flow diagram of the literature search and the selection of the study.

According to our first database search, 272 items were identified (**Figure 1**). An initial literature search through the PubMed, Embase, Web of Science, and Cochrane database databases yielded 265 published articles after duplicates were removed. When reviewed by titles or abstracts,187 records did not meet the inclusion criteria, leaving 88 potentially relevant studies that were reviewed in full text. Among the remaining 88 articles, 2 were reviews, 16 were meta-analyzes and 2 were meeting conferences; these publications were also excluded. Left 58 publications were left, 2 were insufficient data, 4 were overlapping data, and 8 were not in HWE (**Tables 1**–**3**). Finally, a total of 44 publications were included in the meta-analysis, among which 9 case-control studies from 9 publications with 4111 cases and 2669 controls for the RAD51 G172T polymorphism, and 20 case-control studies from 11 publications with 20183 cases and 20321 controls for the XRCC2 R188H polymorphism and a total of 47 studies from 38 publications with 26667 cases and 27912 controls for the XRCC3 T241M polymorphism were eventually included in our meta-analysis. We checked the symmetry of the Begg funnel plot and the results of Egger’s test to assess publication bias. All statistical analyzes were performed with STATA version 16.0.

**Table 1:** Main characteristics of all studies included in the meta-analysis of the RAD51 G172T polymorphism.

Author [Reference]	Year	Source of control	Ethnicity	Method	Case			Control			HWE	NOS score
					GG	GT	TT	GG	GT	TT		
**Kuschel B**	2002	PB	Caucasian	Taqman	744	1061	430	226	371	139	0.54	6
**Lee**	2005	HB	Asian	PCR	721	54	9	533	54	4	0.05	6
**Silva**	2009	HB	Caucasian	TaqMan	94	139	55	168	275	105	0.69	6
**Vral**	2011	PB	Caucasian	PCR-RFLP	36	34	30	50	81	23	0.29	2
**Sassi**	2013	HB	Caucasian	PCR-RLFP/ PCR-CTPP	13	152	139	0	59	260	0.07	5
**Michalska**	2015	HB	Caucasian	PCR-RFLP	17	11	42	20	40	10	0.16	6
**Al Zoubi**	2015	PB	Arab	Sequencing	22	14	70	17	29	8	0.44	6
**Al Zoubi**	2017	PB	Caucasian	PCR-RFLP	5	3	14	6	9	1	0.32	6
**Al Zoubi**	2021	HB	Arab	sequencing	66	83	53	68	87	26	0.83	6

PB: population-based; HB: hospital-based; HWE: Hardy-Weinberg equilibrium (significant at the 0.05 level) ; NOS: The Newcastle-Ottawa Scale, Quality of studies based on NOS star scoring system: 1–2 stars: poor, 3–5 stars: fair and 6–10 stars: good)

For overlapping studies, only the one with the largest sample numbers was included for meta-analysis.

**Table 2:** Main characteristics of all studies included in the meta-analysis of the XRCC2 R188H polymorphism.

Author [Reference]	Year	Source of control	Ethnicity	Method	Case			Control			HWE	NOS score
					GG	GA	AA	GG	GA	AA		
**Millikan-1**	2002	PB	African Americans	Taqman	744	21	0	653	25	0	0.63	9
**Millikan-2**	2002	PB	Caucasian	Taqman	1084	176	8	982	145	7	0.52	9
**Han**	2004	NA	Caucasian (99%)	TaqMan/ABI PRISM	811	134	7	1066	165	6	0.89	8
**BCAC HBCCS**	2006	HB	Caucasian (German)	PCR-RFLP	222	31	1	161	32	1	0.66	5
**BCAC LSHTM**	2006	PB	Caucasian	PCR-RFLP	491	91	3	507	84	7	0.11	6
**BCAC Madrid**	2006	HB	Caucasian (Spanish)	Taqman	695	152	16	698	136	11	0.14	7
**BCAC US3-state**	2006	PB	Caucasian	Taqman	1662	198	5	1117	1214	11	0.71	7
**BCAC SEARCH**	2006	PB	Caucasian (98%)	Taqman	3698	638	32	4385	824	37	0.80	6
**BCAC Sheffield**	2006	PB	Caucasian	Taqman	818	145	10	807	155	6	0.62	7
**BCAC PBSC**	2006	PB	Caucasian (Polish)	Taqman	1305	234	10	1983	281	16	0.08	7
**BCAC USRTS**	2006	HB	Mixed	Taqman	587	122	3	882	161	3	0.12	7
**Brooks**	2008	NA	Mixed	PCR-RFLP	515	83	4	519	78	5	0.28	8
**Webb-1**	2008	PB	Mixed	ABI PRISM	1251	187	9	675	101	7	0.15	8
**Webb-2**	2008	PB	Caucasian	ABI PRISM	1113	177	8	562	90	6	0.26	8
**Romanowicz-Makowska**	2012	NA	Caucasian	PCR-RFLP	182	344	174	172	376	160	0.09	7
**Qureshi**	2014	PB	Asian	PCR	131	20	5	137	20	1	0.21	6
**Ding**	2015	HB	Asian	PCR-LDR	166	280	160	184	305	144	0.41	7
**Smolarz**	2015	HB	Caucasian	PCR-RFLP	12	8	50	18	40	12	0.21	6
**Shadrina**	2016	PB	Caucasian	Taqman	594	65	0	587	67	2	0.95	6
**Rajagopal**	2022	PB	Asian	PCR-RFLP	376	106	9	394	95	4	0.51	7

PB: populaion-based: hospital based; NA: not available; HWE: Hardy-Weinberg equilibrium (significant at the 0.05 level); NOS: The Newcastle-Ottawa Scale, Quality of studies based on NOS star scoring system: 1–2 stars: poor, 3–5 stars: fair and 6–10 stars: good)

For overlapping studies, only the one with the largest sample number was included in the meta-analysis.

**Table 3:** Main characteristics of all studies included in the meta-analysis of the XRCC3 T241M polymorphism.

Author [Reference]	Year	Source of control	Ethnicity	Method	Case			Control			HWE	NOS score
					TT	TM	MM	TT	TM	MM		
**Millikan**	2002	PB	African Americans	Taqman	505	578	101	435	555	142	0.09	9
**Rafii S**	2002	HB	Caucasian	Taqman	201	248	72	341	416	169	0.87	8
**Smith a**	2003	HB	Caucasian	PCR-RFLP	96	105	51	104	129	35	0.61	7
**Smith b**	2003	PB	Caucasian	PCR-RFLP	62	74	26	112	141	49	0.68	7
**Jacobsen**	2003	PB	Caucasian	Taq-Man / PCR-RFLP	163	203	59	160	198	65	0.77	4
**Forsti**	2004	PB	Caucasian	PCR-RFLP	72	85	15	89	88	25	0.65	4
**Han**	2004	NA	Caucasian (99%)	TaqMan/ABI PRISM	388	429	135	468	607	170	0.23	8
**Figueiredo**	2004	HB	Caucasian (99%)	MALDI-TOF MS	139	186	77	146	200	56	0.34	8
**Zhang**	2005	HB	Asian	PCR-RFLP	33	80	107	29	115	166	0.17	3
**Thyagarajan**	2006	PB	Caucasian	PCR-RFLP	160	192	27	126	157	40	0.41	8
**BCAC HBCCS**	2006	HB	Caucasian (German)	Taq-Man & ARMS	95	119	42	77	88	29	0.64	5
**BCAC SEARCH**	2006	PB	Caucasian (98%)	Taqman	1177	1462	405	1607	1898	549	0.76	6
**BCAC Sheffield**	2006	PB	Caucasian	Taqman	458	555	168	437	534	195	0.14	7
**BCAC USRTS**	2006	HB	Mixed	Taqman	281	336	98	402	480	155	0.55	7
**Garcia-Closas-1**	2006	PB	Caucasian	Taqman	1102	1419	457	973	1213	368	0.75	7
**Garcia-Closas-2**	2006	PB	Caucasian	Taqman	785	907	282	980	1039	266	0.71	7
**Sangrajrang**	2007	HB	Asian	Melting curve analysis	437	69	1	384	38	2	0.32	6
**Lee**	2007	PB	Asian	Single base extension assay	437	51	1	349	29	0	0.74	6
**Brooks**	2008	NA	Mixed	PCR-RFLP	254	259	98	249	286	76	0.31	8
**Webb-1**	2008	PB	Mixed	ABI PRISM	591	656	198	307	375	106	0.61	8
**Webb-2**	2008	PB	Caucasian	ABI PRISM	500	612	184	248	321	91	0.43	8
**Loizidou**	2008	PB	Mixed	PCR-RFLP	312	560	220	351	600	226	0.29	8
**Sobczuk**	2009	HB	Caucasian	PCR-RFLP	29	71	50	24	50	32	0.57	5
**Sterpone**	2010	HB	Caucasian	PCR-RFLP	18	21	4	15	15	4	0.85	6
**Santos**	2010	HB	Mixed	PCR-RFLP	28	31	6	49	29	7	0.37	6
**Jara**	2010	PB	Mixed	CSGE	149	91	27	296	182	22	0.52	7
**Silva**	2010	HB	Caucasian	PCR-RFLP	109	138	42	178	276	94	0.46	6
**Vral**	2011	HB	Caucasian	PCR-RFLP or SnapShot technique	60	87	23	54	84	30	0.96	2
**Gonzalez-Hormazabal**	2012	HB	Mixed	Taqman	187	103	32	335	209	23	0.18	7
**Romanowicz-Makowska**	2012	PB	Caucasian	PCR-RFLP	190	348	162	158	354	960	0.94	7
**Ramadan**	2014	HB	Mixed	PCR-RFLP	28	57	15	30	37	38	0.49	7
**Qureshi**	2014	PB	Asian	PCR	74	67	15	101	44	5	>0.05	6
**Ding **	2015	HB	Asian	PCR-LDR	510	91	5	557	74	2	0.25	7
**Su**	2015	HB	Asian	PCR-RFLP	1052	141	39	1131	87	14	0.89	7
**Smolarz**	2015	HB	Caucasian	PCR-RFLP	19	35	16	15	35	20	0.72	6
**Lavanya**	2015	HB	Asian	PCR-RFLP	42	7	1	40	8	2	>0.05	6
**Al Zoubi**	2015	HB	Arab	Sequencing	16	26	4	8	18	5	0.33	5
**Shadrina**	2016	PB	Caucasian	Taqman	285	284	95	294	278	72	0.59	5
**Al Zoubi**	2017	HB	Caucasian	Sequencing	8	13	2	4	9	2	0.72	5
**Kipen**	2017	HB	Caucasian	PCR-RFLP	86	68	15	84	94	7	>0.05	5
**Devi**	2017	PB	Asian	PCR-RFLP	350	100	14	426	99	9	0.25	9
**Ozgoz**	2017	HB	Mixed	ultiplex-PCR & MALDI-TOF	42	46	14	37	40	23	0.23	6
**Howlader**	2020	HB	Asian	PCR-RFLP	70	46	5	96	34	3	0.99	6
**Rajagopal**	2022	PB	Asian	PCR-RFLP	310	158	23	342	134	17	0.39	7

PB: population-based; HB: hospital-based; NA: not available; HWE: Hardy-Weinberg equilibrium (significant at the 0.05 level); NOS: The Newcastle-Ottawa Scale, Quality of studies based on NOS star scoring system: 1–2 stars: poor, 3–5 stars: fair and 6–10 stars: good)

For overlapping studies, only the one with the largest sample numbers was included in meta-analysis.

### Meta-analysis result

**Figure 2:**
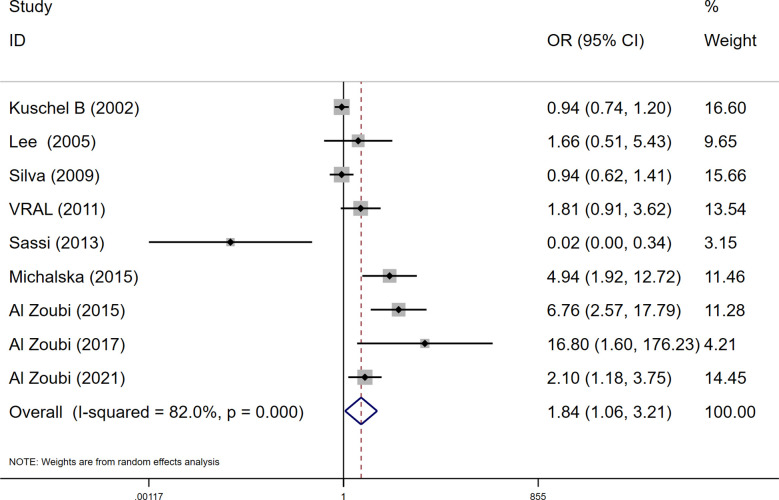
Meta-analysis of the RAD51 G172T polymorphism and risk in breast cancer (homozygote model, TT vs. GG).

**Figure 3:**
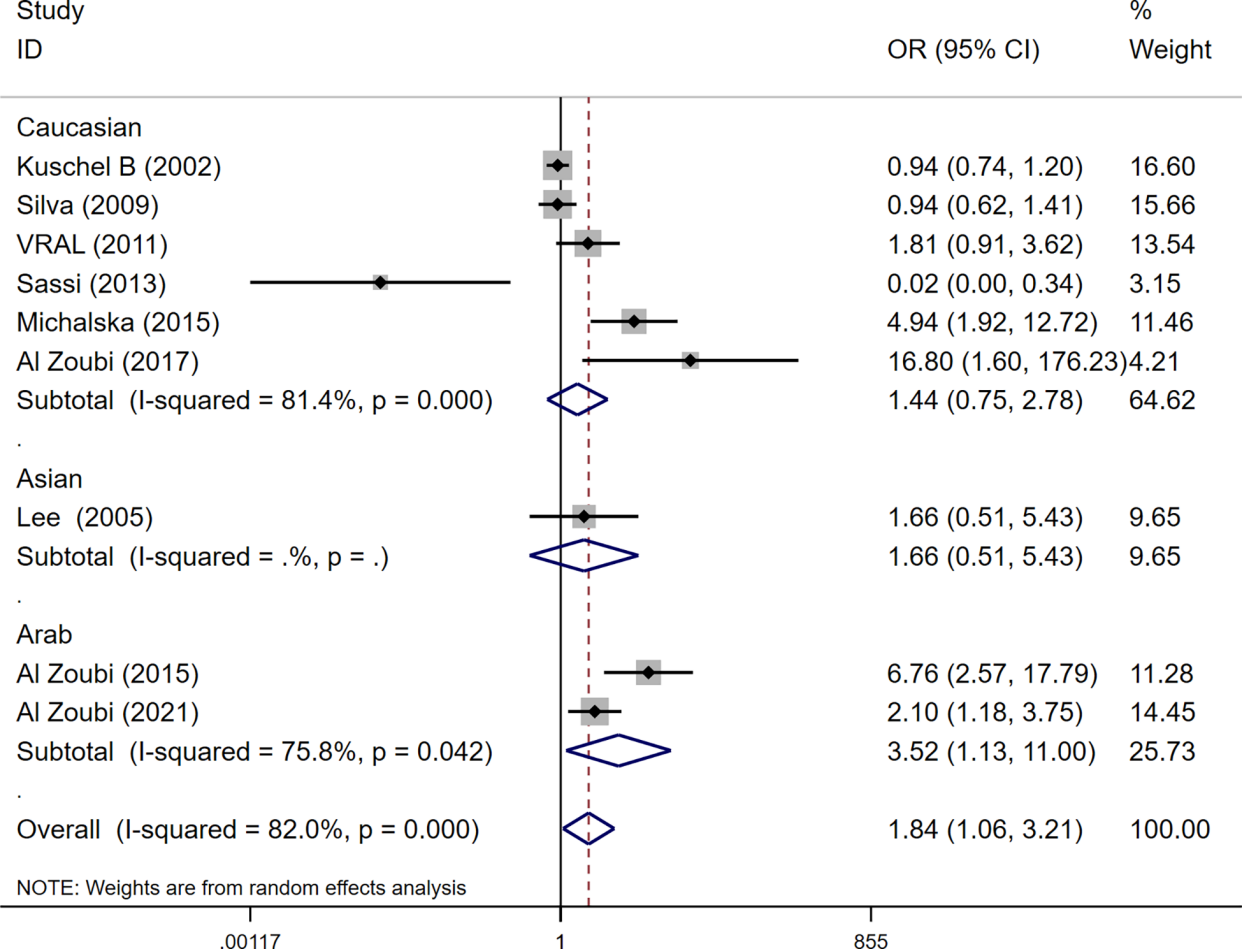
Subgroup analysis according to the ethnicity of the RAD51 G172T polymorphism (homozygote model, TT vs. GG).

Among these 9 case-control studies from 9 publications with 4111 cases and 2669 controls for the RAD51 G172T polymorphism (32–40). The combined results showed that there was no significant correlation between the G172T polymorphism and breast cancer susceptibility in all genetic models except the homozygote model (homozygote model: OR = 1.84, 95% CI = 1.06-3.21, **Figure 2**; dominant model: OR = 0.97, 95% CI = 0.80–1.18; recessive model: OR = 0.47, 95% CI = 0.22–1.00; allelic genetic model: OR = 1.15, 95% CI = 0.79–1.68). Additionally, ethnic-based analysis showed that SNP was associated with breast cancer risk in Arab populations in homozygous models (OR=3.52, 95% CI=1.13-11.0, *P*= 0.003) (**Figure 3**). It suggests that the G172T polymorphism may be associated with an increased risk of breast cancer in the Arab population in some cases. When stratified by the source of controls, our results found evidence of an association between cancer risk and the G172T polymorphism in population-based controls in the recessive model (OR=0.25, 95% CI=0.07-0.85, *P*= 0.027), suggesting that it is marginally related to the population-based group.

For the R188H polymorphism XRCC2, 20 case-control studies from 11 articles with 20183 cases and 20321 controls for the XRCC2 R188H polymorphism (41–51). No significant association was observed between this polymorphism and breast cancer susceptibility (homozygote model: OR = 1.13, 95% CI = 0.88–1.46; dominant model: OR = 1.01, 95% CI = 0.92-1.11, recessive model: OR = 0.83, 95% CI = 0.61-1.12; allelic genetic model: OR = 1.05, 95% CI = 0.95-1.17.).

**Figure 4:**
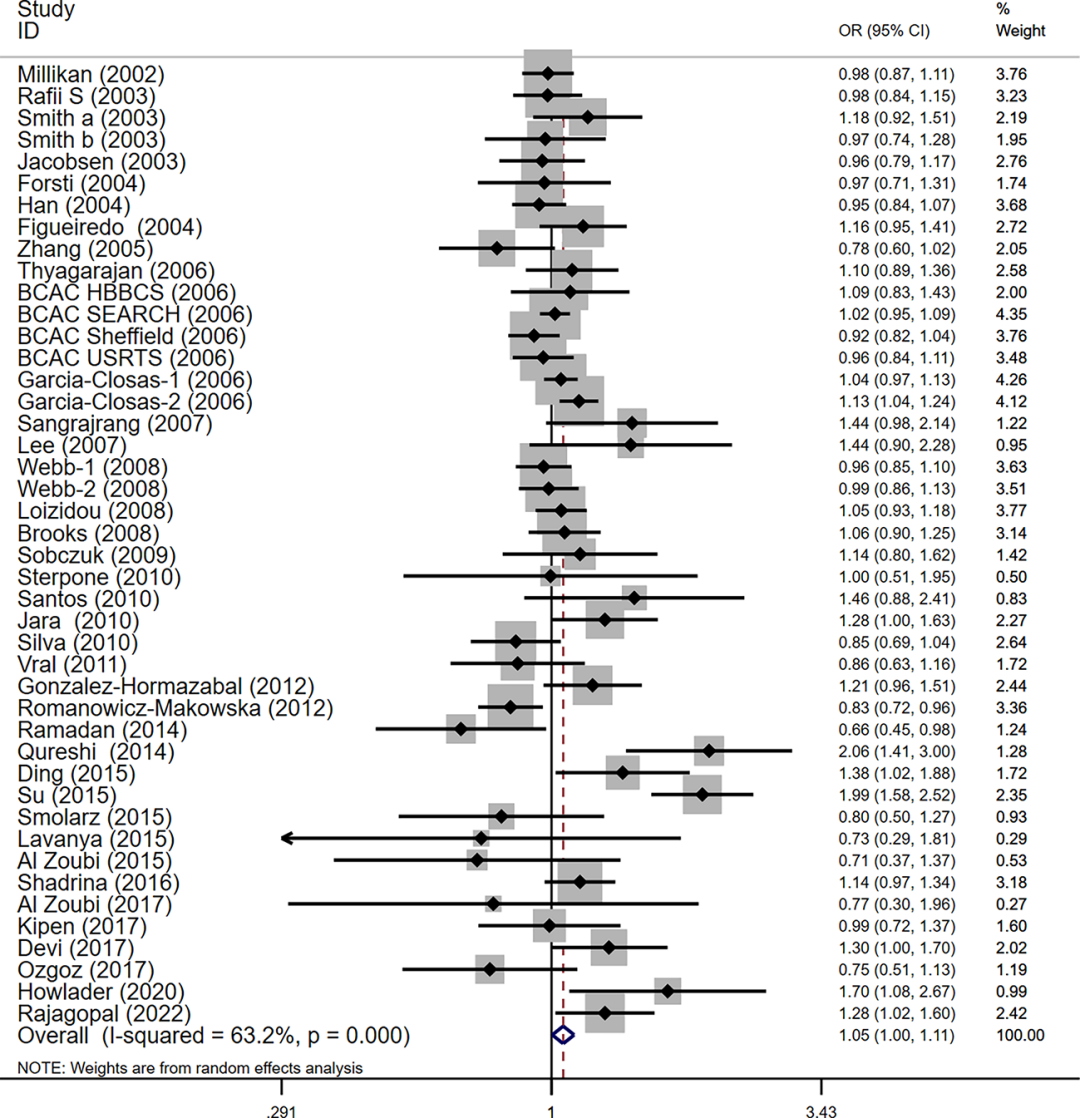
Forest plots of the XRCC3 T241M polymorphism and risk of breast cancer (allelic genetic model, M vs. T).

For the polymorphism in XRCC3 Thr241Met, a total of 47 studies of 38 articles with 26667 cases and 27912 controls were eventually included in our meta-analysis (32, 37, 38, 44, 45, 47, 48, 52–80). A significant increase in cancer risk was observed only in the allelic genetic model (homozygote model: OR = 1.08, 95% CI = 0.98–1.20; dominant model: OR = 1.05, 95% CI = 0.99–1.12; recessive model: OR = 0.92, 95% CI = 0.84–1.01; allelic genetic model: OR = 1.05, 95% CI = 1.00–1.11) (**Figure 4**). In addition, ethnic-based analysis showed that SNP was associated with breast cancer risk in Asian populations in dominant genetic (OR = 1.36,95% CI= 1.11–1.66, *P* = 0.003) and allelic genetic models (OR = 1.32,95% CI 1.07–1.64, *P* = 0.01) (**Tables 4**, **5**).

**Table 4:** Meta-analysis of the Rad51 G172T polymorphism on the risk of breast cancer.

Analysis model	Homozygote model	heterogeneity		Dominant model	heterogeneity		Recessive model	heterogeneity		Allelic genetic models	heterogeneity	
	OR(95%CI) *P*	*P*h	I^2^	OR(95% CI) *P*	*P*h	I^2^	OR(95% CI) P	*P*h	*I* ^2^	OR(95%CI)P	*P*h	*I* ^2^
**Total**	1.84 (1.06,3.21)0.031*	0.000	82%	0.97 (0.80,1.20)0.77	0.123	37%	0.47 (0.22,1.00)0.05	0.000	95.1%	1.15 (0.79,1.68)0.459	0.000	93.2%
Ethnicity												
**Caucasian**	1.44 (0.75,2.78)0.28	0.000	81.4%	0.92 (0.72,1.16)0.46	0.196	32%	0.60 (0.24,1.49)0.27	0.000	95.9%	0.97 (0.61,1.53)0.885	0.000	93.7%
**Arab**	3.52 (1.13,11.00)0.03*	0.042	75.%	1.35 (0.94,1.95)0.11	0.425	0%	0.21 (0.04,1.09)0.06	0.001	90.7%	2.24 (0.87,5.79)0.095	0.001	94.1%
Source of control												
**PB**	2.71 (0.95,7.71)0.062	0.000	86.5%	1.00 (0.74,1.36)0.98	0.228	30.8%	0.25 (0.07,0.85)0.027*	0.000	92.7%	1.80 (0.95,3.41)0.07	0.000	91.2%
**HB**	1.39 (0.57,3.35)0.469	0.000	81.9%	0.95 (0.68,1.32)0.76	0.076	52.7%	0.72 (0.21,2.46)0.599	0.000	96.3%	0.85 (0.46,1.56)0.598	0.000	94.8%

PB: population-based; HB: hospital-based; HWE: Hardy-Weinberg equilibrium (significant at the 0.05 level);

a P-values for ORs; Ph values of the Q-test for heterogeneity test; I^2^ refers to the proportion of total variation due to between-study heterogeneity;

^b* mark^ means the positive results.

c Random-effects model was used when the Ph value for the heterogeneity test was <0.05; otherwise, the fixed effects model was used.

**Table 5:** Meta-analysis of the XRCC3 T241M polymorphism on the risk of breast cancer.

Analysis model	Homozygote model	heterogeneity		Dominant model	heterogeneity		Recessive model	heterogeneity		Allelic genetic models	heterogeneity	
	OR (95%CI) *P*	*P*h	*I* ^2^	OR (95% CI) *P*	*P*h	*I* ^2^	OR (95% CI) *P*	*Ph*	*I* ^2^	OR (95%CI)*P*	*P*h	*I* ^2^
**Total**	1.08 (0.98,1.20) 0.125	0.000	54.3%	1.05 (0.99,1.12)0.09	0.000	50.1%	0.92 (0.84,1.01)0.09	0.000	55.3%	1.06 (1.00,1.12)0.04*	0.000	63.2%
Ethnicity												
**Caucasian**	1.03 (0.94,1.13) 0.578	0.037	36.9%	1.00 (0.96,1/05)0.87	0.577	0.0%	0.96 (0.88,1.05)0.36	0.04	36.3%	1.01 (0.97,1.05)0.78	0.145	23.7%
**Asian**	1.45 (0.83,2.55) 0.193	0.013	58.8%	1.36 (1.11,1.66)0..003*	0.013	58.6%	0.69 (0.43,1.10)0.12	0.044	49.7%	1.32 (1.07,1.64)0.01*	0.000	73.8%
**Mixed**	1.20 (0.91,1.60) 0.203	0.000	72.7%	1.09 (0.94,1.27)0.30	0.044	48.0%	0.88 (0.65,1.17)0.37	0.000	78.2%	1.07 (0.95,1.21)0.26	0.001	68.8%
Source of control												
**PB**	1.07 (0.96,1.20)0.23	0.002	55.9%	1.04 (0.97,1.11)0.24	0.021	44.7%	0.94 (0.85,1.04)0.22	0.008	50.3%	1.04 (0.99,1.10)0.15	0.001	59.8%
**HB**	1.08 (0.86,1.36)0.495	0.000	57.5%	1.09 (0.96,1.23)0.19	0.001	53.4%	0.93 (0.75,1.15)0.50	0.000	61.6%	1.07 (0.96,1.19)0.25	0.000	67.7%
**NA**	1.07 (0.82,1.40)0.60	0.212	35.9%	0.91 (0.79,1.04)0.17	0.497	0.0%	0.8 (0.68,1.10)0.24	0.222	33.0%	0.370	N	N

PB: population-based; HB: hospital-based; HWE: Hardy-Weinberg equilibrium (significant at the 0.05 level); NA: not available

a *P*-values for ORs; *P*h values of the Q-test for heterogeneity test; *I* ^2^ refers to the proportion of total variation due to between-study heterogeneity

* refers to P<0.05 and had a statistical significance.

### Prognostic factors

**Figure 5:**
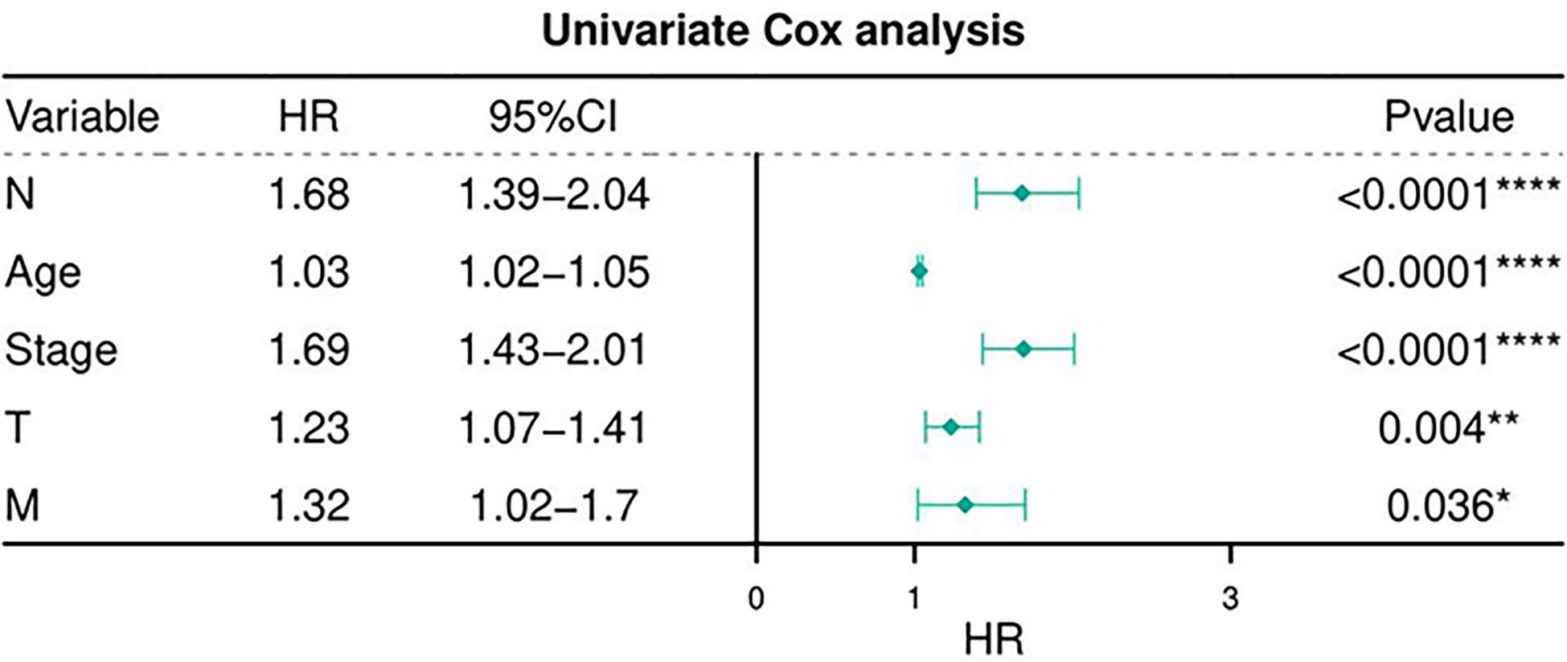
Univariate Cox regression analyses of OS in BC patients.

**Figure 6:**
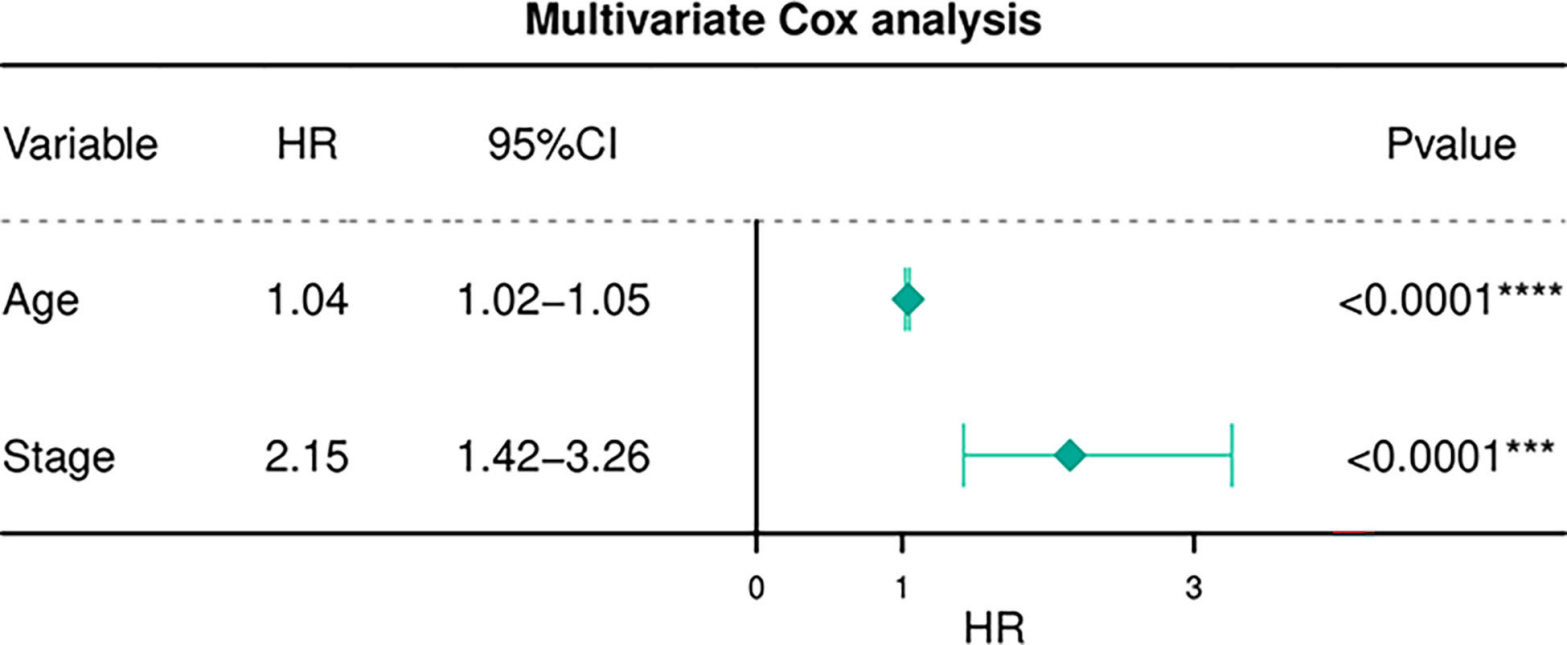
Multivariate Cox regression analyses of OS in BC patients.

**Table 6** depicts the pooled results from the univariable and the multivariable analyses of OS in BC patients (HR). Univariate and multivariate Cox regression analysis was performed to determine whether gene expression is an independent prognostic model of OS in breast cancer patients. As shown in **Figure 5**, the p values of T, N, M, Stage, and Age were less than 0.05. The results of the univariate Cox regression analysis of OS showed that pathology stage, age, and stage could effectively predict survival in BC patients. Then, we took these factors into the multivariate Cox regression analysis. Furthermore, after the multivariate analyses (**Figure 6**), the results showed that stage (HR =2.15; 95% CI, 1.42−3.26), age (HR = 1.04; 95% CI, 1.02-1.05) remained independent prognostic factors with an adjusted P value < 0.0001.

**Table 6:** RAD51 univariate Cox regression analyses of OS in BC patients.

Clinicopathologic parameters	OS					
	Univariate Cox analysis			Multivariate Cox analysis		
	HR	95% CI	P value	HR	95% CI	P value
Age	1.03	1.02-1.05	<0.0001****	1.04	1.02-1.05	<0.0001****
M	1.32	1.02-1.7	0.036*	0.88	0.62-1.26	0.495
N	1.68	1.39-2.04	<0.0001****	1.09	0.81-1.46	0.577
RAD51	2.65	0.37-19.01	0.334			
Stage	1.69	1.43-2.01	<0.0001****	2.15	1.42-3.26	<0.0001***
T	1.23	1.07-1.41	0.004**	0.97	0.82-1.15	0.747
XRCC2	0	0-Inf	0.994			

OS: overall survival, HR: Hazard ratio.

Only one mutated sample in XRCC3, but there was no survival information, it was rounded off in the analysis.

P value < 0.05 was considered significant. The asterisk ( * ) indicates p < 0.05 ; two asterisks ( * * ) represent p < 0.01, and four asterisks ( * * * * ) represent p < 0.0001.

### Sensitive analysis

Given the significant heterogeneity between studies for the polymorphisms, the random-effect model was used to calculate the pooled results if the heterogeneity was significant. Meanwhile, we also performed a sensitivity analysis to assess the effects of each study on the pooled ORs by omission of individual studies. The sensitivity analysis showed that, for each polymorphism, no single study qualitatively changed the pooled ORs, suggesting that the results of this meta-analysis were statistically stable and reliable.

### Publication bias diagnostics

We further identify potential publication biases of the literature using the Egger test and funnel plot. In all studies, no funnel plot asymmetry was found. The results of Egger’s test for the RAD51 G172T polymorphism did not show any evidence of publication bias. For the homozygote model, the funnel plot p-value was 0.47, and Egger’s test p-value was 0.185. In the dominant model, Begg’s test results of the R188H *P* value were 0.67, and Egger’s test *P* value was 0.319. Begg’s test result of the allelic genetic model in XRCC3 T241M *P* = 0.65 and Egger’s test result showed *P* = 0.52, suggesting no publication bias. All *P*-values> 0.05, suggesting that there was no publication bias.

## Discussion

Screening for some frequent polymorphisms has improved our understanding of the critical roles that inheritance plays in BC susceptibility. To date, associations between genetic variants in HRR genes and BC development have been investigated, but the results remain unexplained to the best of our knowledge. However, new discoveries in drug research aimed at these gene mutations are always innovative. Some experiments suggest that the inhibition of HR will be selective against breast tumor cells. Inhibitors of HR proteins can be used in combination with radiotherapy or chemotherapy to sensitize the cells [5]. A more intriguing possibility would be to use anti-HR agents alone, avoiding the toxicity of DNA-damaging agents. Such a strategy has been applied to selectively kill BRCA2-deficient cells using poly-ADP-ribose-polymerase inhibitors (PARP). The first phase III clinical study of PARP inhibitor for adjuvant treatment of early breast cancer, OlympiA study, aims to evaluate the efficacy and safety of olaparib compared with placebo in the adjuvant treatment of early breast cancer with clinically and pathologically high-risk, HER2-negative, BRCA1/2 mutation. And randomized phase II GeparOLA study showed olaparib plus paclitaxel (PO) in early HER2-negative homologous recombination deficiency (HRD) breast cancer. In conclusion, germline BRCA 1/2 status and HRD predict a higher pathological complete response (pCR) rate in the neoadjuvant treatment (81). The molecular mechanism of breast cancer is very complex. Therefore, in the post-PARP inhibitor era, there is a great clinical need to find therapeutic targets and analyze prognostic factors to benefit patients, which is conducive to drug development and expansion of new indications and provides the possibility of individualized treatment for breast cancer.

Our analysis demonstrated the importance of recombination repair processes for the fidelity of chromosome segregation and reinforce the functional connection between genes involved in HRR and those that predispose to breast cancer. We also found that patients in our prediction models tended to be older, have an advanced-stage disease, and have a poorer prognosis. Current literature varies widely in experimental methods, stage of disease, family history of cancer, patients with the type of tumor therapy, and the duration since cancer diagnosis, all of which can lead to inconsistent results in case-control studies. Additionally, most of the studies did not specify the immunohistochemical indicators of breast cancer that are relevant to determine which factors can exert a dominating effect. Some research data indicate that double-strand break damage is the most fatal lesion observed in eukaryotic cells because it can cause cell death or create a serious threat to cell viability and genome stability. It has the potential to permanently arrest cell cycle progression and endanger cell survival [10]. Due to the fact that DNA repair mechanisms are crucial to preserving genomic stability and functionality, DNA repair defects can result in the development of chromosomal aberrations that can lead to increased susceptibility to cancer (4, 82, 83). A Japanese study showed that Rad51 gene polymorphisms were found in two patients with bilateral breast cancer (10). It proves that germline mutations in the RAD51 gene may modulate the risk of breast cancer. Previous meta-analyses evaluated the effect of the Rad51 G135C polymorphism on the risk of breast cancer and other cancers. Some experts performed relevant meta-analyses of the analysis and concluded that the Rad51 G172T polymorphism may play a protective role in the development of head and neck cancer, but no significant correlation was found between the Rad51 G172T polymorphism and breast and ovarian cancer (84). It is inconsistent with our conclusion and hypothesized that it was related to inadequate inclusion of the sample size, neglecting gene-gene and gene-environment interactions for some reason. However, there were some approvals on the connection of polymorphism in XRCC2 R188H and the risk of breast cancer before, which has not been confirmed in two population studies in the United States and Poland and several case-control experimental studies (39, 42–44, 50, 51, 68). Moreover, an experiment conducted by RafiiS was hardly replicated in the latest BCAC study (41). Several studies describe a marginally protective effect for rare allele carriers (188His) (64, 85). Interestingly, Silva suggested that the potential protective role of the variant allele of XRCC2, in women who have never breastfed, could be related to a more efficient DNA repair activity (37). On the other hand, Han described a protective effect for women with high plasma α-carotene levels. However, current evidence shows that in most studies the XRCC2 R188H polymorphism is considered to have little relationship with the risk of breast cancer. According to our meta-analysis of breast cancer, we did not find a significant association between this polymorphism and breast cancer susceptibility, which is consistent with the previous meta-analysis. In previous studies, a relevant study reported their results with significant unexplained heterogeneity (*P*h = 0.014) (86). Furthermore, studies that depart from the Hardy-Weinberg equilibrium (HWE) were included in the meta-analysis, which may lead to potential bias. Current evidence suggests that XRCC2 R188H polymorphism is considered to have a weak protective effect against breast cancer development in most studies, but the association did not reach statistical significance. As we mentioned above, since this effect is very weak and R188H may serve as a positional marker for other potentially functional SNPs or haplotypes, it is not surprising that this SNP is not associated with breast cancer, or even in an inverse relationship. Therefore, limited by the above factors, the interpretation of the results of previous research should be cautious. A common polymorphism in the XRCC3 gene is at nucleotide 1,8607C/T which results in the substitution of the amino acid threonine for methionine at codon 241 (Thr241Met) of exon 7 of the XRCC3 gene, which may affect the function of the encoding enzyme or/and its interaction with other proteins involved in DNA repair. Inheritance of functional polymorphisms in DNA repair genes may influence the capacity of the DNA repair process, thus leading to increased cancer risk. Due to a C18607T transition at exon 7 of the XRCC3 gene, the substitution of amino acids Thr241Met is functionally active, as it is associated with an increase in the number of micronuclei in human lymphocytes exposed to ionizing radiation (59, 67, 72, 87, 88). The variant allele (241Met) is associated with high levels of DNA adducts in lymphocyte DNA, which could be associated with reduced DNA repair capacity (88). A case-control study in Pakistan found that homozygous (TT) and heterozygous (TM) genotypes of the T241M polymorphism were associated with an increased risk of breast cancer compared to controls (47). Similar results have previously been observed in different studies, suggesting an association between Met allele variants and breast cancer in Caucasian and Asian populations (63, 65). Interestingly, Rajagopal found that heterozygous genotype (TM) and homozygous mutant genotype (MM) were not significantly associated with breast cancer risk when it comes to the role of the T241M variation in XRCC3 (48). Chai performed a meta-analysis of 23 case-control studies on the association of XRCC3 SNPs with the risk of breast cancer in the above SNPs and the general population and the Asian population in both recessive and homozygous models (89). Our results based on racial stratification analysis are consistent with their observed correlations in Asian populations, but not the same with their associated models. Although they found an association between this SNP and the risk of sporadic breast cancer, based on the conclusive results obtained, we believe that this association is not accurate enough. Although other studies have not shown an association between T241M polymorphism and the risk of breast cancer (52, 54). Therefore, more studies are needed to confirm these associations.

Compared with studies before, our study has some improvements. First, Our study had the advantage of including higher numbers of cases and controls. Second, these polymorphisms in RAD51 and paralog genes were analyzed and associated with the risk of specific cancer, breast cancer. Third, we provided a more comprehensive analysis of the data by calculating four different genetic models and performing a subgroup analysis by ethnicity, and source of controls (population or hospital-based). Finally, we excluded studies in which the distribution of genotypes in the control group was inconsistent with HWE because they might influence the results. The results of this study further revealed the correlation between the polymorphism in these genes and the occurrence and development of breast cancer, providing a direction for the study of molecular mechanisms of cancer in the future.

The main limitations of our meta-analysis are: 1) This meta-analysis only searched published studies in English, ignoring some unpublished studies or studies in other languages that may also meet the inclusion criteria. 2) Some studies did not provide enough clinical data such as patient family history, ER/PR, HER-2 hormone receptor status, tissue type, and tumor grade, leading to failure to conduct a comprehensive subgroup analysis to explore the source of heterogeneity. 3) Gene-gene and gene-environment interactions were not considered in current meta-analyses. Possible gene-gene and gene-environment interactions between Rad51 gene polymorphism and cancer susceptibility need to be further studied. 4) some patients were chosen from hospital-based groups, and these women may have benign breast disease, corresponding to an increased potential risk of breast cancer. 5) Most of the patients in our study were Caucasian, which may limit the general application of our results.

## Data availability statement

The original contributions presented in the study are included in the article/**Supplementary Material**. Further inquiries can be directed to the corresponding author.

## Author contributions

All authors contributed to the study’s conception and design. Material preparation, data collection, and analysis were performed by JY and C-GW. Manuscript drafting and reviewing: All authors. All authors contributed to the article and approved the submitted version.
