# Supplementary material for: Relationship between polymorphisms in homologous recombination repair genes RAD51 G172T、XRCC2 & XRCC3 and risk of breast cancer: A meta-analysis
Source: Front Oncol. 2023 Jan 24;13:1047336. doi: 10.3389/fonc.2023.1047336 (PMC9903134; doi:10.3389/fonc.2023.1047336)
Supplement: Supplementary file 1 [file Table_1.docx]

Table S1 NEWCASTLE-OTTAWA QUALITY ASSESSMENT SCALE FOR CASE-CONTROL STUDIES INCLUDED IN META-ANALYSIS

| Author [Reference] | Selection | | | | Comparability of Controls on the Basis of the Design or Analysis^a^ |  | Exposure | | | NOS Scores |
| --- | --- | --- | --- | --- | --- | --- | --- | --- | --- | --- |
|  | Adequate definition of cases | Representativeness of the cases | Selection of controls | Definition of controls |  |  | Assessment of exposure | Some methods of Assessment for cases and controls | non-response rate |  |
| Kuschel B 2002 | ☆ | ☆ | ☆ | ☆ | ☆ |  | - | ☆ | - | 6 |
| Millikan-1 2002 | ☆ | ☆ | ☆ | ☆ | ☆☆ |  | ☆ | ☆ | ☆ | 9 |
| Millikan-2 2002 | ☆ | ☆ | ☆ | ☆ | ☆☆ |  | ☆ | ☆ | ☆ | 9 |
| Rafii S 2002 | ☆ | ☆ | - | ☆ | ☆☆ |  | ☆ | ☆ | ☆ | 8 |
| Smith a 2003 | ☆ | ☆ | - | ☆ | ☆☆ |  | ☆ | ☆ | - | 7 |
| Smith b 2003 | ☆ | ☆ | ☆ | ☆ | ☆ |  | ☆ | ☆ | - | 7 |
| Jacobsen 2003 | - | ☆ | ☆ | ☆ | - |  | - | ☆ | - | 4 |
| Forsti 2004 | ☆ | - | ☆ | - | - |  | ☆ | ☆ | - | 4 |
| Han 2004 | ☆ | ☆ | - | ☆ | ☆☆ |  | ☆ | ☆ | ☆ | 8 |
| Figueiredo 2004 | ☆ | ☆ | - | ☆ | ☆☆ |  | ☆ | ☆ | ☆ | 8 |
| Lee 2005 | ☆ | ☆ | - | ☆ | ☆☆ |  | - | ☆ | - | 7 |
| Zhang 2005 | - | ☆ | - | - | ☆ |  | - | ☆ | - | 3 |
| Thyagarajan 2006 | ☆ | ☆ | ☆ | ☆ | ☆☆ |  | ☆ | ☆ | - | 8 |
| BCAC LSHTM 2006 | ☆ | ☆ | ☆ | ☆ | ☆ |  | - | ☆ | - | 6 |
| BCAC Madrid 2006 | ☆ | ☆ | - | ☆ | ☆☆ |  | - | ☆ | - | 7 |
| BCAC US3-state 2006 | ☆ | ☆ | ☆ | ☆ | ☆☆ |  | - | ☆ | - | 7 |
| BCAC PBSC 2006 | ☆ | ☆ | ☆ | ☆ | ☆☆ |  | - | ☆ | - | 7 |
| BCAC HBCCS 2006 | ☆ | ☆ | - | ☆ | ☆ |  | - | ☆ | - | 5 |
| BCAC SEARCH 2006 | ☆ | ☆ | ☆ | ☆ | ☆ |  | - | ☆ | - | 6 |
| BCAC Sheffield 2006 | ☆ | ☆ | ☆ | ☆☆ | ☆☆ |  | - | ☆ | - | 7 |
| BCAC USRTS 2006 | ☆ | ☆ | ☆ | ☆ | ☆☆ |  | - | ☆ | - | 7 |
| Garcia-Closas-1 2006 | ☆ | ☆ | ☆ | ☆ | ☆☆ |  | - | ☆ | - | 7 |
| Garcia-Closas-2 2006 | ☆ | ☆ | ☆ | ☆ | ☆☆ |  | - | ☆ | - | 7 |
| Sangrajrang 2007 | ☆ | ☆ | - | ☆ | ☆☆ |  | - | ☆ | - | 6 |
| Lee 2007 | ☆ | ☆ | ☆ | ☆ | ☆ |  | - | ☆ | - | 6 |
| Brooks 2008 | ☆ | ☆ | - | ☆ | ☆☆ |  | ☆ | ☆ | ☆ | 8 |
| Webb-1 2008 | ☆ | ☆ | ☆ | ☆ | ☆☆ |  | ☆ | ☆ | - | 8 |
| Webb-2 2008 | ☆ | ☆ | ☆ | ☆ | ☆☆ |  | ☆ | ☆ | - | 8 |
| Loizidou 2008 | ☆ | ☆ | ☆ | ☆ | ☆☆ |  | ☆ | ☆ | - | 8 |
| Sobczuk 2009 | ☆ | ☆ | - | ☆ | ☆ |  | - | ☆ | - | 5 |
| Sterpone 2010 | ☆ | ☆ | - | ☆ | ☆ |  | ☆ | ☆ | - | 6 |
| Santos 2010 | ☆ | ☆ | - | ☆ | ☆☆ |  | - | ☆ | - | 6 |
| Jara 2010 | ☆ | ☆ | ☆ | ☆ | ☆ |  | ☆ | ☆ | - | 7 |
| Silva 2010 | ☆ | ☆ | - | ☆ | ☆ |  | - | ☆ | ☆ | 6 |
| Vral 2011 | - | ☆ | - | - | - |  | - | ☆ | - | 2 |
| Gonzalez-Hormazabal 2012 | ☆ | ☆ | - | ☆ | ☆☆ |  | ☆ | ☆ | - | 7 |
| Romanowicz-Makowska 2012 | ☆ | ☆ | ☆ | ☆ | ☆☆ |  | - | ☆ | - | 7 |
| Sassi 2013 | ☆ | ☆ | - | ☆ | ☆ |  | - | ☆ | - | 5 |
| Ramadan 2014 | ☆ | ☆ | - | ☆ | ☆☆ |  | - | ☆ | - | 7 |
| Qureshi 2014 | ☆ | ☆ | ☆ | ☆ | ☆ |  | - | ☆ | - | 6 |
| Michalska 2015 | ☆ | ☆ | - | ☆ | ☆☆ |  | - | ☆ | - | 6 |
| Ding 2015 | ☆ | ☆ | - | ☆ | ☆☆ |  | ☆ | ☆ | - | 7 |
| Su 2015 | ☆ | ☆ | - | ☆ | ☆☆ |  | ☆ | ☆ | - | 7 |
| Smolarz 2015 | ☆ | ☆ | - | ☆ | ☆ ☆ |  | - | ☆ | - | 6 |
| Lavanya 2015 | ☆ | ☆ | - | ☆ | ☆ |  | - | ☆ | - | 6 |
| Al Zoubi 2015 | ☆ | ☆ | ☆ | ☆ | ☆ |  | - | ☆ | - | 6 |
| Shadrina 2016 | ☆ | ☆ | ☆ | ☆ | ☆ |  | - | ☆ | - | 6 |
| Al Zoubi 2017 | ☆ | ☆ | ☆ | ☆ | ☆ |  | - | ☆ | - | 6 |
| Kipen 2017 | ☆ | ☆ | - | ☆ | ☆ |  | - | ☆ | - | 5 |
| Devi 2017 | ☆ | ☆ | ☆ | ☆ | ☆☆ |  | ☆ | ☆ | ☆ | 9 |
| Ozgoz 2017 | ☆ | ☆ | - | ☆ | ☆☆ |  | ☆ | ☆ | - | 7 |
| Howlader 2020 | ☆ | ☆ | - | ☆ | ☆☆ |  | - | ☆ | - | 6 |
| Al Zoubi  2021 | ☆ | ☆ | - | ☆ | ☆☆ |  | - | ☆ | - | 6 |
| Rajagopal 2022 | ☆ | ☆ | ☆ | ☆ | ☆ |  | ☆ | ☆ | - | 7 |

NOS: The Newcastle-Ottawa Scale, Quality of studies based on NOS star scoring system: 1–2 stars: poor, 3–5 stars: fair, and 6–10 stars: good)
